# Uppsala Biobank—the development of a biobank organization in a local, regional, and national setting

**DOI:** 10.1080/03009734.2018.1547992

**Published:** 2019-02-01

**Authors:** Anna Beskow

**Affiliations:** a Department of Immunology Genetics and Pathology, Uppsala University, Uppsala, Sweden;; b Uppsala Biobank, Uppsala Clinical Research Center, Uppsala, Sweden

**Keywords:** Biobank, cancer, infrastructure, organization

## Abstract

A biobank is generally in an international setting considered as a sample collection with linked data. In Sweden we have a lot of sample collections, but the definition of a biobank has changed, and it has become an organization that administrates many sample collections as well as an infrastructure to support research. Uppsala Biobank was started in September 2008 as a joint biobank organization between Uppsala County Council and Uppsala University. At the start there were 138 registered biobanks in Uppsala for these two principals. The decision was to have only one biobank, where all previous biobanks would be transformed to be sample collections. Uppsala Biobank has gone from the wish to centralize biobanking administration to be a research infrastructure, a national model for hospital-integrated biobanking, a support structure for biobanking activities in the health care region, and the local competence center for all biobank issues in Uppsala.

## Background

In 2003 Sweden got its first biobank act, Act (2002: 297) on biobanks in health care. According to that a biobank is defined as a collection of biological material stored for one or more purposes and information on this material. The legislation together with guidelines from the National Board of Health and Welfare (SOSFS 2002: 11 and SOSFS 2004: 2) regulate biobank operations in Sweden. Biobanks may consist of one or more sample collections. The collections of samples have different primary purposes, with collection for health care and diagnostics purposes being most common, followed by collection of samples for research. The Biobank Act applies to human material such as blood, saliva, urine, and different kinds of tissue samples. The donor must give an informed consent that biobank samples are stored and used for approved purposes. To be able to use samples collected within health care for research, an ethical approval and a written consent are needed.

## Collaboration between the Swedish county councils

The legislation was not easily adoptable to general health care and research activities within the health care providers. The need for consent processes and the need to document access to diagnostic samples for research made it difficult for the 21 Swedish counties to set up their own solutions. Unique solutions for each county/health care provider also made it difficult for researchers to understand how to access samples. The Swedish Biobank project started, and it led to the formation of the national Biobank council with a strong national network including six regional biobank centers and 21 biobank coordinators working together.

## Biobanking—a hot topic in the world

Meanwhile, the notion of biobanking was growing globally. The International Society for Biological and Environmental Repositories (ISBER) was formed in 1999 (www.isber.org). Some years later a European chapter was formed (https://esbb.org/). The European Commission acknowledged that the competition globally in medical research using biobanks was increasing. Large countries such as USA and China with larger populations for biobanking had an advantage that the European countries had to face together. The preparatory phase of BBMRI (ESFRI roadmap) started in 2008 with the goal to form an ERIC to make it possible for researchers to collaborate within the EU to increase the population base.

## Start of Uppsala Biobank

In 2007 a prospect for a common biobank between Karolinska Institutet and Stockholm County Council—Stockholm Biobank—was presented. The idea to have a joint biobank was that this would make it easier for the users, who usually work for both principals, to join resources and work more efficiently together. Stockholm Biobank as it was then presented has not yet been formed, even though efforts have been made. The idea was picked up by professors Christer Sundström and Ulf Gyllensten in Uppsala. They convinced Uppsala Academic Hospital and the Medical Faculty at Uppsala University to join forces and start Uppsala Biobank to become the single joint human biobank. Uppsala Biobank started on 1 September 2008 and had four aims: 1) to administrate Uppsala Biobank; 2) to build a stable and long-term organization; 3) to be a competence center for biobank matters; 4) and to build an infrastructure for research.

## Administration of Uppsala Biobank

The first decision of the local Biobank council was that there would be only one biobank in Uppsala, which meant transforming all previous 138 biobanks into sample collections. It took four years for one person to perform this task, working half-time (1). Now Uppsala Biobank consists of approximately 170 research sample collections and eight sample collections for diagnostic purposes (e.g. clinical pathology, clinical microbiology, and clinical genetics). Uppsala Biobank administrates about 100 biobank applications every year. On average half of them are applications to access samples from clinical pathology, and more than half are applications to collect and access samples for clinical trials. Less than 10% become new sample collections. Each sample collection has a responsible person linked with a written agreement between the director of the biobank and the person responsible for the sample collection. The agreement includes rights and responsibilities. The rights give the person responsible for the sample collection the disposition right to the samples and clarify the responsibility regarding safe keeping of the samples and data to secure quality and integrity according to the legislation.

## A stable and long-term organization

There is a signed agreement between the principals, Region Uppsala represented by Uppsala Academic Hospital and Uppsala University represented by the Medical Faculty. The agreement describes the organization, mission, responsibilities, finances, and steering of Uppsala Biobank. Each principal equally funds Uppsala Biobank. Region Uppsala is the legal principal of the samples, and Uppsala University is the research principal.

## A competence center for biobank matters

Uppsala Biobank activities include information and education of researchers, staff, and students. Questions are either sent to the biobank by e-mail or telephoned. In 2017 more than 500 questions were received, and they ranged across the whole spectrum of biobanking. Uppsala Biobank has a web page that was released in a new and updated version in April 2017 (www.uppsalabiobank.uu.se).

## An infrastructure for research

In 2009 the Swedish Government announced investments in strategic research projects and infrastructures. Uppsala University filed several applications including biobank activities. Together with Umeå University, Uppsala University applied for a cancer research infrastructure: Uppsala–Umeå comprehensive cancer consortium, U-CAN. The goals of U-CAN include formation of a clinical database and a biobank with samples collected routinely from cancer patients to create the substrate for high-quality cancer research (2). The requirement to collect samples in routine health care was challenging, and there was a need to form collaborations between all actors affected to find a long-term solution. The requirements on the collection of samples were to work in the hands of the routine staff with systems already in use. It also had to be possible to collect samples 24 h a day, seven days a week in order to avoid exclusion of acute cases. Moreover, it must be possible to include not only cancer but also other diagnoses. The solution means that biobank samples can be ordered in the electronic health record as an analysis. When ordering other analyses, a package of biobank samples can be ordered at the same time. Labels are printed, and diagnostic samples and samples for research are taken simultaneously. Samples are sent to the clinical chemistry laboratory, where they are registered, sorted according to sample type, centrifuged and aliquoted with a liquid handler. Samples are frozen and transported to Uppsala Biobank for long-term storage in low-temperature freezers. All information and data about the sample and donor are sent automatically to the Biobank Laboratory Information Management System (Biobank LIMS). The solution is called Health Care Integrated Biobanking (HIB), and now 45 projects collect samples at 77 different clinics. Samples are also taken at primary care centers and sent to the hospital for biobanking.

## The health care region

Health care integrated biobanking has now spread throughout the Uppsala–Örebro health care region. The region includes seven county councils (Uppsala, Örebro, Dalarna, Gävleborg, Värmland, Västmanland, and Sörmland). Apart from Uppsala, also Örebro, Gävleborg, and Värmland have started HIB in 2014, and Dalarna as well as Västmanland will start shortly. Sörmland is planning to start in 2019. The Uppsala–Örebro region collaborates within the regional biobank council. In order to work efficiently the decision was made to share a common regional Biobank LIMS. The regional Biobank LIMS is hosted and developed by Uppsala Biobank. Uppsala Biobank, with the support of the principals, is investing in an automated solution for long-term low-temperature storage (-80 °C). Uppsala Biobank will also support the region with automation for storage and withdrawals if needed, e.g. the county of Gävleborg has decided to send their samples to Uppsala for storage and withdrawals.

## The national contribution

In 2010 the Swedish Research Council founded a national biobank infrastructure called BBMRI.se to be the European counterpart to BBMRI.ERIC. Karolinska Institutet was the host of the infrastructure, which was purely based on university collaboration. The national biobank infrastructure already in place through the national biobank council was a collaboration between all county councils and the universities, but this infrastructure was not included. BBMRI.se aimed to increase the access to samples owned by the county councils, but without including them. Lack of collaboration between the two national infrastructures made the Swedish research council stop the funding of BBMRI.se in 2016. Concurrently, the Uppsala Biobank model of HIB was spread not only to the Uppsala–Örebro health care region but also to the rest of Sweden. Setting up HIB at several hospitals in Sweden has been possible through funding from SweLife and the health care principals to support local and national biobanking projects. Now 19 hospitals in Sweden have HIB in production, and several more are about to start.

Since biobanking is something that the government sees as a strategic area to invest in, the Swedish research council opened up for a new application for a national biobank infrastructure. The requirement was that the principal’s universities with medical faculties and the corresponding county councils collaborated and applied together. In 2016 a joint steering board with the deans from the medical faculties and the research directors from the corresponding county councils was formed to collaborate around the new national biobank infrastructure, Biobank Sweden. The basis for Biobank Sweden comes from the former National Biobank Council with already existing biobanks and network. An application was sent to the Swedish Research Council in March 2017 for funding of some of the development needed of the infrastructure to better support research on biobank samples in Sweden and European collaboration through participation in BBMRI-ERIC. The application was granted funds for 2018–2019.

## Conclusions

In September 2018 Uppsala Biobank celebrates its tenth anniversary. During these 10 years the biobank has been built, starting from 138 separate biobanks with no coordination, to an organization fulfilling all four missions set for the local biobank responsibility. Uppsala Biobank has also taken and takes responsibility for the development of the regional and national biobank infrastructure and has shown that collaboration and openness are important features for success. International collaborations using biobank samples will hopefully further strengthen Swedish research.
